# Icaritin inhibits the expression of alpha-fetoprotein in hepatitis B virus-infected hepatoma cell lines through post-transcriptional regulation

**DOI:** 10.18632/oncotarget.13194

**Published:** 2016-11-08

**Authors:** Chao Zhang, Hui Li, Wei Jiang, Xiaowei Zhang, Gang Li

**Affiliations:** ^1^ Department of Cell Biology and Municipal Laboratory of Liver Protection and Regulation of Regeneration, Capital Medical University, Beijing, China; ^2^ Department of Biochemistry and Molecular Biology, School of Basic Medical Sciences, Peking University Health Science Center, Beijing, China

**Keywords:** alpha-fetoprotein, microRNA, hepatitis B virus, icaritin, post-transcriptional regulation

## Abstract

Although it has showed that icaritin can apparently suppress growth of HCC by reducing the level of AFP, the intrinsic mechanism remains unclear. In this study, we explored the possible mechanism of miRNAs on post-transcriptional regulation of *AFP* gene, as well as the effects of HBV infection and icaritin in hepatoma cells. The results showed that miR-620, miR-1236 and miR-1270 could bind target sites in the range of 9–18 nt and 131–151 nt downstream of the stop codon in the *AFP* mRNA 3′-UTR to suppress the expression of *AFP*. Mutation of these target sites could reverse the effects of these miRNAs. Icaritin (10 μM) might reduce the stability and translational activity of *AFP* mRNA by increasing the expression levels of these mentioned miRNAs. HBV infection resulted in apparent decreases of these miRNAs and, consequently, increased *AFP* expression. The results indicated that miR-620, miR-1236 and miR-1270 are critical factors in the post-transcriptional regulation of *AFP*. Icaritin can counteract the effect of HBV. These findings will contribute to full understanding of the regulatory mechanism of *AFP* expression in hepatoma cells. And also it revealed a synergistic mechanism of HBV infection and elevation of AFP in the pathogenesis of HCC, as well as the potential clinical significance of icaritin on the therapy of HCC induced by HBV.

## INTRODUCTION

Alpha fetoprotein (AFP) is a tumor-associated protein found in certain fetal organs, proliferating hepatocytes and certain adult cancer cells, such as hepatocellular carcinoma (HCC) cells. Circulating AFP acts as a growth regulator during oncogenic growth and tumor progression, and is considered a diagnostic and prognostic tumor marker [1–3]. In recent years, remarkable progress has been made in determining the biological role of cytoplasmic AFP as a signal molecule: aberrantly elevated AFP disturbs the normal signaling network and shows a strong association with the high mortality rate of HCC [4–7].

Cytoplasmic AFP has the ability to disrupt the onward transmission signaling of the RA- RAR and PI3K/AKT signaling, which leads to aberrant growth of hepatocellular carcinoma cells [4, 8]. In addition, the caspase-3 cascade and the tumor necrosis factor (TNF)-related apoptosis inducing ligand (TRAIL) induce apoptosis is virtually abolished in the presence of AFP [5, 9]. Our previous research has shown the potential association of AFP levels with HBV infection and revealed a hitherto undiscovered role for cytoplasmic AFP in mediating HBV-induced hepatocyte carcinogenesis [7]. Given that cytoplasmic AFP has been defined as a growth-promoting molecule, *AFP* gene silencing would be beneficial for therapy of HCC patients. Clinical studies have found that icaritin can reduce the level of AFP to enhance the therapeutic effect of HCC [10]. However, the intrinsic mechanism remains unclear, and in particular, data is insufficient to reveal the relationship between icaritin and HBV in regulating the expression of AFP.

MicroRNAs (miRNAs) inhibit gene expression by binding mainly to the 3′-UTR. Considerable evidence indicates that miRNAs have fundamental roles in development, differentiation, metabolism, growth and apoptosis [11–13]. In HCC, the expressions of many miRNAs are significantly changed [14–18]. These miRNAs have been predicted to serve as promising biochemical markers for HCC diagnosis and may have therapeutic applications in HBV-related HCC.

Icaritin is a traditional Chinese medicine derived from the plant *Herba Epimedium*. It has been confirmed that icaritin can inhibit proliferation and promote apoptosis of a wide variety of tumors, including HCC, by obstructing JAK/STAT3 signaling, and as a consequence, suppressing the expressions of tumor genes such as *BCL-XL, BCL-2, C-MYC* and *SURVIVIN* [10, 19, 20]. In addition, the anti-tumor effects of icariin, which can be hydrolyzed into icaritin, are achieved by regulation of microRNAs that bind to *PTEN* and *RECK* genes [21, 22]. To date, studies regarding icaritin in tumor therapy have mainly focused on signal pathways involved in proliferation or apoptosis. However, detailed research on the mechanisms involved is lacking.

As miRNAs play an important role in the regulation of the expressions of tumor genes, it is important to clarify whether miRNAs mediate post-transcriptional regulation of *AFP* and whether icaritin functions by elevating the level of miRNAs under HBV infection, because HBV infection is directly relevant to AFP elevation in hepatoma cells [7]. Clarification of these mechanisms will provide further insights into the regulation of AFP in hepatoma cells, and offer a new therapy for liver cancer caused by HBV.

## RESULTS

### MiR-620, miR-1236 and miR-1270 inhibit *AFP* mRNA 3′-UTR activity by binding to its target sequences

**Figure 1:**
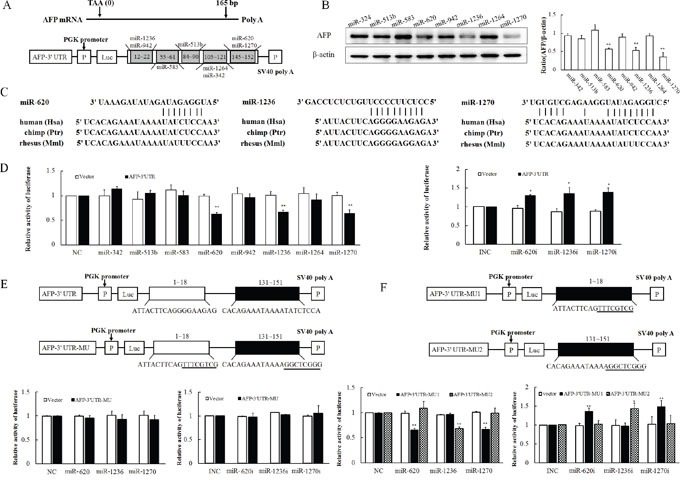
Determination of miR-620, miR-1236, miR-1270 binding sites in the 3′-UTR of the *AFP* mRNA in PLC/PRF/5 cells **A.** Schematic showing potential binding sites of predicted miRNAs in the AFP-3′-UTR construct. **B.** Effect of transfection of various miRNA mimics on the expression of *AFP*. The panel on the right side of images is the densitometric analysis. **C.** Homology analysis of miR-620, miR-1236 and miR-1270 in human, chimp and rhesus. **D.** Relative luciferase activity after transfection of the AFP-3′-UTR construct and miR-620, miR-1236, miR-1270 mimics or inhibitors. **E.** A schematic plot of the luciferase construct AFP-3′-UTR-MU expressing mutational binding sites of miR-620, miR-1236, miR-1270 (upper panel). Relative luciferase activity after co-transfection of AFP-3′-UTR-MU construct and miR-620, miR-1236, miR-1270 mimics (left panel) or inhibitors (right panel). Underlined letters in the schematic represent mutated nucleotides. **F.** Schematic plot of the luciferase constructs AFP-3′-UTR-MU_1_ expressing mutational binding site of miR-1236 and AFP-3′-UTR-MU_2_ expressing mutational binding sites of miR-620, miR-1270 (upper panel). Luciferase activity after co-transfection of AFP-3′-UTR-MU_1_ or AFP-3′-UTR-MU_2_ constructs and miR-620, miR-1236, miR-1270 mimics (left panel) or inhibitors (right panel). These experiments were repeated at least three times. Data represents mean ± SD of three samples. *P < 0.05 and **P < 0.01 as compared with controls.

To define whether predicted microRNAs (miR-324, miR-513b, miR-583, miR-620, miR-942, miR-1236, miR-1264, and miR-1270) (Figure 1A) suppress AFP, eight different miRNAs were transfected into PLC cells. Of these eight miRNAs, only miR-620, miR-1236 and miR-1270 reduced the abundance of the AFP protein (Figure 1B). The numbers in boxes indicate the nucleotide sites downstream from the *AFP* stop codon. The target sites for miR-620, miR-1236 and miR-1270 in the *AFP* 3′-UTR were conserved in different species (Figure 1C). To determine whether miR-620, miR-1236 and miR-1270 suppress AFP through specific binding to the putative 3′-UTR target sites, PLC cells were cotransfected with microRNA mimics and the *AFP*-3′-UTR reporter plasmid respectively. Cotransfection of miR-620, miR1236 and miR-1270 mimics with the *AFP*-3′-UTR caused a ~40% decrease in luciferase activity compared with the control and other microRNAs (Figure 1D left) and co-transfection with their inhibitors led to an apparent increment in luciferase activity of *AFP*-3′-UTR (Figure 1D right). As there are three binding sites for miR-620, miR-1236 and miR-1270 in the *AFP* mRNA 3′-UTR, we mutated all the target sequences in AFP-3′-UTR to generate AFP-3′-UTR-MU. This mutation resulted in significant attenuation of the repressive effect of miR-620, miR-1236 and miR-1270 (Figure 1E). We then generated two mutated constructs corresponding to the individual sites, designated as AFP-3′-UTR-MU1 (containing a mutated miR-1236 target site) and AFP-3′-UTR-MU2 (containing mutated miR-620 and miR-1270 target sites). Co-transfection of certain mutated constructs with miR-620, miR-1236 and miR-1270 mimics and inhibitors led to corresponding decreases and increases in the luciferase activity (Figure 1F). These results suggested there are sequence-specific interactions between miR-620, miR-1236 and miR-1270 and their binding sites in the *AFP* mRNA 3′-UTR.

### MiR-620, miR-1236, miR-1270 inhibit expression of AFP

**Figure 2:**
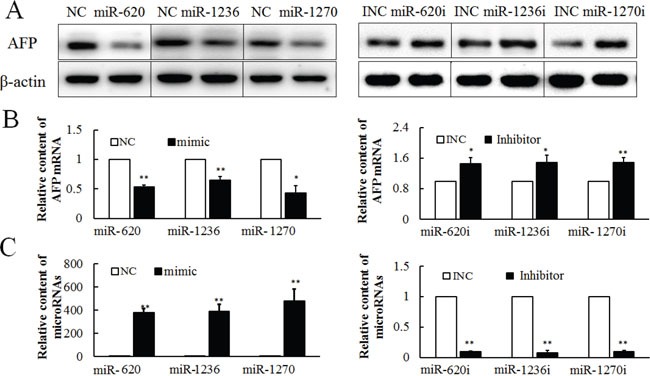
Effect of miR-620, miR-1236 and miR-1270 on the expression of AFP in PLC/PRF/5 cells, as evaluated by western blotting or qRT-PCR Effect of miR-620, miR-1236 and miR-1270 mimic or inhibitor on AFP protein A. and mRNA B. at 36 h after transfection. **C.** The effect of hsa-miR-620, miR-1236, miR-1270 mimics (50 pmol/well) or inhibitors (50 pmol/well) on miR-620, miR-1236, miR-1270 levels at 36 h after transfection. NC: Negative control for miR-620, miR-1236, miR-1270 mimic. Inhibitor NC or INC: Negative control for the inhibitor. These experiments were repeated at least three times. Each image is representative of three independent experiments. Data represents mean ± SD of three samples. **P <0.01 as compared with controls.

To determine the potential role of miRNAs in the regulation of *AFP* expression, miR-620, miR-1236 and miR-1270 were transfected into PLC cells for 36 h. All three miRNAs could reduce the content of AFP protein (Figure 2A left). The effect of miR-620, miR-1236 and miR-1270 on *AFP* expression was further validated using their inhibitors, which increased AFP expression (Figure 2A right). All the western bloting images were analyzed by gray scale analysis (Supplementary Figure S1). As expected, transfection with miR-620, miR-1236 and miR-1270 mimics suppressed the expression of *AFP* mRNA, which was reversed by transfection with miR-620, miR-1236 and miR-1270 inhibitors (Figure 2B). The inhibitors of miR-620, miR-1236 and miR-1270 specifically reduced the levels of endogenous miR-620, miR-1236 and miR-1270 (Figure 2C). The specificity of the inhibitors of miR-620, miR-1236 and miR-1270 were further confirmed by observation of the effects of the inhibitors on various miRNAs (Supplementary Figure S2).

### The role of icaritin on expression of miR-620, miR-1236, miR-1270 and AFP

**Figure 3:**
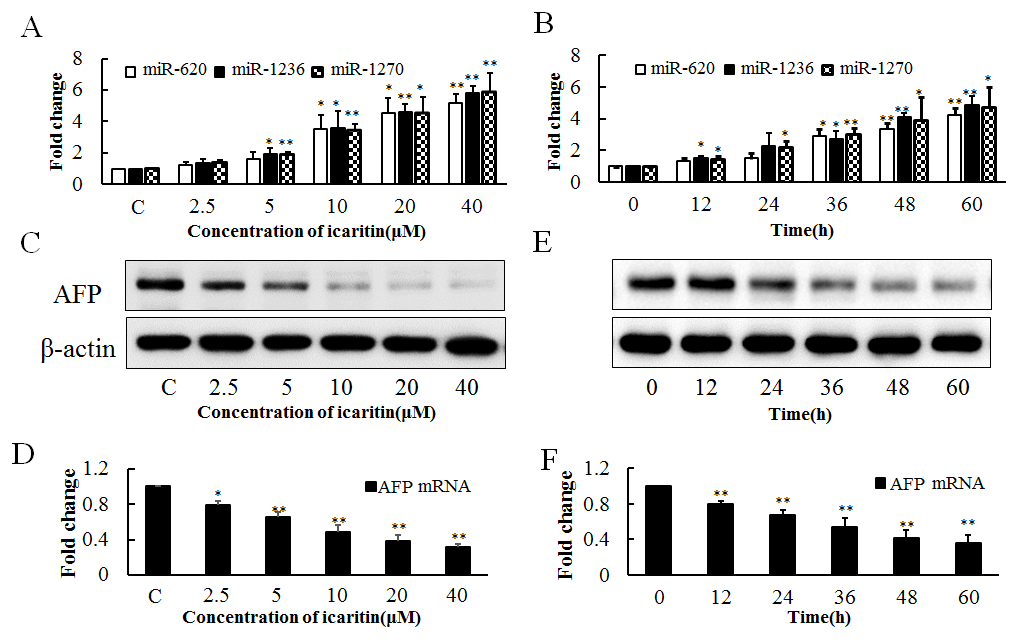
Effects of icaritin on miR-620, miR-1236, miR-1270 and *AFP* expression in PLC/PRF/5 cells **A.** Effects of different concentrations of icaritin (2.5–40 μM) on the expressions of miR-620, miR-1236, miR-1270 at 48 h of treatment. **B.** Effect of icaritin (10 μM) on the expressions of miR-620, miR-1236, miR-1270 at different time points (12–60 h). Effect of different concentrations of icaritin on AFP protein **C**. and *AFP* mRNA **D**. Effect of different incubation time of icaritin on AFP protein **E**. and *AFP* mRNA **F**. The image is representative of three independent experiments. Data represents mean ± SD of three samples. *P< 0.05 and **P< 0.01 as compared with controls.

To verify the effect of icaritin on the expressions of endogenous miR-620, miR-1236, miR-1270 and AFP, PLC cells were treated with various concentrations of icaritin (2.5–40 μM). The results showed that icaritin increased the expressions of miR-620, miR-1236 and miR-1270 (Figure 3A) and inhibited the expression of AFP at the protein and mRNA levels (Figures 3C and 3D). The stimulative role of icaritin on miR-620, miR-1236 and miR-1270 was also observed at different treatment times (0–60 h) (Figure 3B). Icaritin decreased the level of AFP from 12 – 60 h (Figures 3E and 3F). Thus icaritin might reduce *AFP* expression by inhibiting the transcription of *AFP* and promoting the degradation of its mRNA. All the western bloting images were analyzed by gray scale analysis (Supplementary Figure S3).

### Icaritin inhibits proliferation of PLC/PRF/5 cells

**Figure 4:**
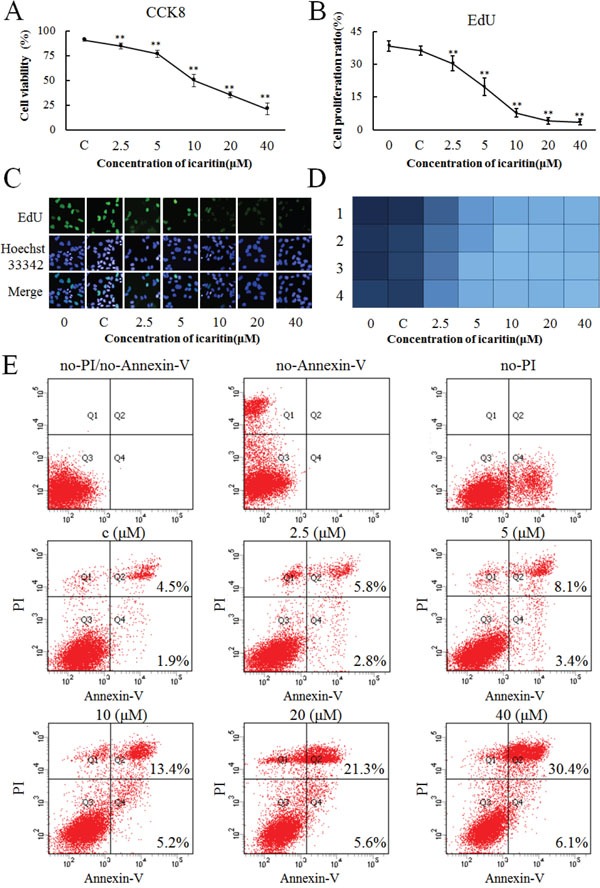
Effects of icaritin on proliferation of PLC/PRF/5 cells **A.** Different concentrations (C, 2.5, 5, 10, 20 and 40 μM/L) of icaritin were tested in cell culture. The viability of PLC/PRF/5 cells was evaluated using a cell counting kit (CCK)-8 assays at 48 h. **B.** The proliferation of PLC/PRF/5 cells was evaluated by an EdU assays after treatment with different concentrations of icaritin at 48 h. **C.** Heat map of PLC/PRF/5 cell proliferation after treatment with different concentrations of icaritin at 48 h. A deeper color represents more cell proliferation. **D.** The proliferation of different concentrations of icaritin-treated PLC/PRF/5 cells captured under a high content imaging system. Proliferating cells are labeled with EdU (Green) and nuclei are stained with Hoechst33342 (Blue). The images are representative of the results obtained. Data are representative of experiments that were repeated three times and are presented as mean ± SD for 6–9 samples. *P < 0.05 and **P < 0.01 as compared with controls. **E**. The apoptosis of different concertrations of icaritin-treated PLC/PRF/5 cells analyzed by flow cytometry.

To further determine the effect of icaritin on hepatoma cell proliferation, CCK-8 and EdU cell proliferation assays were used. As icaritin suppresses AFP expression, it was conceivable that icaritin could counteract the role of AFP in promoting proliferation. CCK-8 and EdU assays were performed after treatment with icaritin (2.5–40 μM) in PLC cells (AFP positive) for 48 h. Cell viability gradually decreased as the effect of AFP was counteracted by increasing doses of icaritin (Figure 4A). However, icaritin had little effect on the growth of L02 normal human hepatocytes (Supplementary Figure S4). At the same time, with increasing icaritin dosage, the cell proliferation rate declined significantly and rapidly (Figure 4B). As shown by the high content imaging system, the inhibitory effect of icaritin increased significantly with the increasing concentration (Figure 4C). Heat maps of the cell proliferation rate were produced by the high content imaging system, in which a deeper color represents more cell proliferation and the four rows (1, 2, 3 and 4) of the graph indicate the results from four parallel samples (Figure 4D). To further confirm the effect of icaritin on apoptosis, PLC cells were treated with different concentrations of icaritin (0–40 μM) for 48 h. Flow cytometric analysis showed that icaritin caused an elevation in the percentage of apoptotic cells (Figure 4E). Compared with the group treated with DMSO, the percentages of apoptotic cells were obviously increased to 8.6% (2.5μM icaritin), 11.5% (5μM icaritin), 18.6% (10μM icaritin), 26.9% (20μM icaritin) and 36.5% (40μM icaritin).

### HBV infection inhibits miR-620, miR-1236, miR-1270 transcription

**Figure 5:**
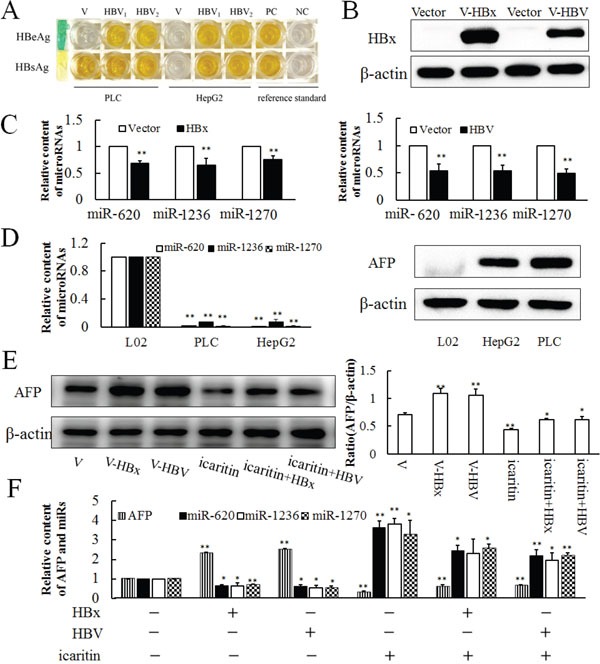
Expressions of miR-620, miR-1236, miR-1270 in HBV- and HBx-transfected cells **A.** HBsAg and HBeAg were detected in the cell culture medium by ELISA after 36 h of infection with HBV in PLC and HepG2 cells. PC. Positive control. NC. Negative control. **B.** Western blotting analysis of HBx and HBV transfected cell lines. **C.** Effects of HBV and HBx transfection on miR-620, miR-1236, miR-1270 expressions after 36 h. **D.** Background expressions of miR-620, miR-1236, miR-1270 and AFP in L02, PLC and HepG2 cell lines analyzed by qRT-PCR and western blotting. **E.** Effect of HBV and HBx infection cooperate with icaritin on AFP expression analysis by western blotting (E) and qRT-PCR **F**. The panel on the right side of the images is the densitometric analysis. The each image is representative of at least three independent experiments. Data represents mean ± SD of three samples. *P < 0.05 and **P < 0.01 as compared with controls.

Previous studies confirmed that HBV and HBx could promote the expression of *AFP*. To determine whether these effects function via post-transcriptional regulation or transcriptional regulation, we sampled HBV and HBx constructs transfected PLC cells to analyze their miR-620, miR-1236, miR-1270 and AFP levels. After transfection of HBV for 36 h in PLC and HepG2 cells, HBsAg and HBeAg were detected in the cell culture medium by ELISA (Figure 5A), and both the HBV and HBx constructs could express HBx in HepG2 and PLC cells, compared with non-transfected ones (Figure 5B). HBV and HBx transfection led to significant decreases in miR-620, miR-1236 and miR-1270 levels (Figure 5C); However, they had no effect on the expression of miR-342, miR-513b, miR-583, miR-942 and miR-1264 (Supplementary Figure S5). At the same time, compared with normal liver cells (L02), the background values of miR-620, miR-1236 and miR-1270 of hepatoma cells (PLC and HepG2) were significantly reduced (Figure 5D). The effects of HBV and HBx on *AFP* expression were reversed by icaritin treatment (Figure 5E and 5F). Also, opposite effects of HBV, HBx and icaritin on miR-620, miR-1236 and miR-1270 levels were observed (Figure 5F). The inverse correlation of reduced miR-620, miR-1236 and miR-1270 and elevated AFP gene expression under viral loading suggested strongly that HBV affected post-transcriptional regulation of the *AFP* gene in PLC cells. The results also suggested that icaritin might have some utility for the treatment of HBV-induced hepatoma.

## DISCUSSION

HCC is the fifth most common malignant neoplasm in the world, whose incidence is rising year by year. Each year, about 500 000–600 000 people die from HCC, and it has become the third most frequent cause of cancer-related death worldwide [2, 23–25]. Clinical studies have shown that the level of serum AFP is associated with a series of malignant characteristics of hepatocellular carcinoma [26, 27]. Furthermore, disease-free survival and overall survival are negatively correlated with serum AFP (>10 ng/mL) [26, 28, 29]. In our previous research, intracellular AFP was shown to be a signal molecule. Recently, a number of proteins, including nuclear receptors and other intracellular signal molecules involved in cell growth or apoptosis, have been reported to bind to cytoplasmic AFP [4, 5, 7, 30]. These results indicated that the poor prognosis associated with high APF is caused by high cell proliferation, high angiogenesis and low apoptosis. Considering the important functions of AFP, in this study, we explored an alternative way of treating HCC by reducing AFP.

HBV is the major epidemiological risk factor for HCC. The attributable fraction of HCC caused by HBV infection ranges from 16% in the USA to 65% in China and the Far East [31, 32]. Compared with non-infected populations, the lifetime risk of developing HCC is 10- to 25-fold greater for chronic HBV carriers. In addition, HBV cirrhotic patients with rising AFP levels were at very high risk of HCC development. [33–35]. Our early work demonstrated the stimulatory effect of HBV on the *AFP* mRNA 5′-UTR, and HBV infection is one of main explanations for elevated AFP levels and the incidence of HCC. Thus, we could easily discover that there is a significant association between HBV infection, AFP rise and hepatocarcinogenesis. The reduction of AFP levels caused by icaritin reversed the effect of HBV, which provides a paradigm for the treatment of HCC.

Over the past few years, significant alterations in the miRNA expression profiles between Hepatitis B Virus infection, HCC and nontumor tissue have been demonstrated by many studies. Several miRNAs have been predicted to affect the initiation and progression of HCC, which might represent a new approach to study the molecular mechanisms, diagnosis, and implementation of novel therapeutic targets in HCC, especially HBV-related HCC [15–17, 36]. Opinions vary as to whether miRNAs have important functions in HCC. Besides, whether the *AFP* gene is post-transcriptionally regulated in hepatoma cells remains unknown. The functions of miRNAs in *AFP* gene expression in hepatoma cells, as well as a correlation with disease and therapy, have not yet been reported.

In our study, we found that HBV infection decreased the expressions of miR-620, miR-1236 and miR-1270 significantly, and compared with normal liver cells (L02), the background expressions of these three miRNAs were obviously reduced in hepatoma cells. MiR-620, miR-1236 and miR-1270 could bind to the 3′-UTR region of *AFP*, and the interaction was disrupted by mutations of bases in the seed sequence. These sites of the 3′-UTR are conserved across species, including humans, chimpanzees and rhesus monkeys (Figure 1C). Overexpression of miR-620, miR-1236 and miR-1270 in hepatoma cell line was associated with an incremental increase in binding to the specific sites of *AFP* mRNA 3′-UTR, as well as a decrease in AFP. The reverse situation was observed in the hepatoma cell line in which endogenous miR-620, miR-1236 and miR-1270 were diminished using a small interfering RNA. The present results, together with our previous work on the regulation of the 5′-UTR, showed that an increase in *AFP* gene expression resulted from transcriptional and post transcriptional gene regulation during the course of HBV infection promotes cell malignant proliferation, ultimately accelerating the process of carcinogenesis.

Although there have been many reports concerning the inhibition of tumor cell proliferation by icaritin, the specific mechanism is unclear. Previous studies reported that icaritin activates the JNK signaling pathway to promote apoptosis in hepatoma cells, and inhibits the IL-6/Jak2/Stat3 pathway to suppress HCC initiation and malignant growth by inducing the expression of anti-apoptotic factors of the Bcl-2 family [10, 37]. In addition, icaritin reverses multidrug resistance (MDR) of HepG2/ADR human hepatoma cells, which was verified by the decrease in MDR1 and P-glycoprotein (P-gp) expressions [38]. More details about the role of icaritin in inhibiting tumor cell proliferation have been published. Icaritin triggers the mitochondrial/caspase apoptotic pathway, by decreasing the Bcl-2/Bax protein ratio and increasing activation of caspase-3 in SMMC-7721 hepatoma cells [39]. Icaritin can also induce cell cycle arrest at the S phase, sustain the phosphorylation of ERK and p38 MAPK, and reduce c-Myc and MMP expressions to inhibit proliferation and promote apoptosis of various tumor cells [40, 41]. Furthermore, icaritin has little toxicity in normal hepatocytes compared with Cisplatin and has little effect on the growth and apoptosis of L02 human hepatocytes. Clinical studies showed that icaritin is very safe, even after oral administration of 1600 mg per day [10, 37]. These results implied that icaritin might possess selective antitumor effects. Previous studies regarding icaritin as a tumor therapy mainly focused on several signaling pathways involved in proliferation or apoptosis. However, there has been little research on the influence of icaritin on miRNAs.

The current study showed that icaritin could counteract the role of HBV to inhibit the expression of AFP by promoting the expressions of miR-620, miR-1236 and miR-1270 in a dose- and time-dependent manner. Considering the promotion effect of AFP on cell proliferation via escape from immune surveillance and binding to signal molecules involved in growth or apoptotic pathways, icaritin can suppress proliferation and accelerate apoptosis as a direct result of inhibiting AFP production. Similarly, the icaritin-induced growth inhibition and apoptosis were time-and dose-dependent. This speculation was confirmed by the results of this study. Although there are few reports regarding to the role of miR-620, miR-1236 and miR-1270 and their relationship with icaritin, our findings provide the missing part of the jigsaw.

**Figure 6:**
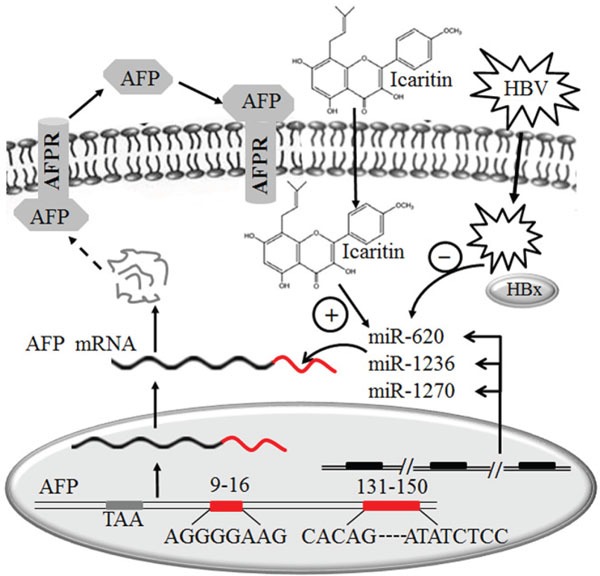
Schematic model of the possible role of miR-620, miR-1236 and miR-1270 in the post-transcription inhibition of *AFP* expression in hepatoma cells and the effects of icaritin and HBV on these miRNAs

Collectively, our findings indicated that decreases in the levels of miR-620, miR-1236 and miR-1270 resulting from HBV infection lead to elevation of AFP levels. Icaritin can inhibit the elevation of AFP by promoting the expression of the three miRNAs, thereby delaying the hepatocellular growth and tumorigenesis. Our data address the role of the three miRNAs in posttranscriptional regulatory mechanisms which is schematically outlined in Figure 6. The clarification of this intrinsic mechanism will further our understanding of the clinical significance of elevated AFP in HBV-induced HCC. The development of a potential therapeutic strategy through the role of icaritin in elevation of specific miRNAs and therefore blocking AFP expression holds promise for the effective control of HCC. Results from this study will be of clinical significance in reduction the incidence of HCC in patients with hepatitis B cirrhosis and high AFP expression. and be helpful for full understanding of the precise mechanism of icaritin on the therapy of HCC.

## MATERIALS AND METHODS

### Prediction of miRNAs and target sites in the 3′-UTR of the AFP gene

UCSC (http://genome.ucsc.edu/) and GenBank were used to obtain the *AFP* 3′-UTR sequence. Websites: RNA22 (https://cm.jefferson.edu/rna22/), RNAhybrid (http://bibiserv.techfak.uni-bielefeld.de/rnahybrid/) and MicroInspector were used to predict miRNAs targeting the 3′-UTR of the *AFP* mRNA [42]. Putative target sites for miR-620, miR-1236 and miR-1270 were predicted using RegRNA (http://regrna.mbc.nctu.edu.tw/html/tutorial.html), PITA (http://genie.weizmann.ac.il/pubs/mir07/mir07_prediction.html), RNAhybrid and RNA22 [43].

### Cell culture

HepG2, PLC cells are both AFP-positive human hepatocellular carcinoma cells. L02 cells (a normal human liver cell line that produces no detectable AFP) was purchased from ShangHai MeiXuan biological science and technology Ltd. All cells were maintained in High Glucose Dulbecco modified Eagle medium (DMEM) medium supplemented with 10% fetal calf serum (FCS) at 37°C in a humidified atmosphere with 5% CO_2_. The PLC and HepG2 cell lines are highly susceptible to HBV infection and icaritin, and express AFP protein at a high levle.

### Icaritin treatment and detection of miR-620, miR-1236, miR-1270 and AFP

To examine the effects of different concentrations of icaritin on transcription of *AFP*, cells (2×10^5^ cells per ml) were added to 6-well plates and treated with different concentrations of icaritin (2.5–40 μM). Icaritin was dissolved in DMSO. After 48 h of incubation, total cellular RNA was extracted from cell lines using the TRIzol reagent (Thermo, Waltham, WA, USA), according to the manufacturer's instructions. To evaluate the effects of icaritin incubation time, icaritin (10 μM) was used to treat the cells and harvested at various time intervals from 0 to 60 h. Routine RNA extraction was then performed. Detection of miR-620, miR-1236 and miR-1270 was carried out by quantitative real-time PCR after reverse transcription using a miRcute miRNA First-Strand cDNA Synthesis Kit (Beijing ComWin Biotech, China). U6 was used as reference control for mature miR-620, miR-1236, miR-1270 and β-actin was used as control for *AFP* with primers as listed in Supplementary Table S1 (Supplementary Table S1) [43].

### Plasmid transfection

pZac2.1-HBV, which can transcribe and assemble HBV in host cells, was a gift from the Department of Microbiology, Peking University Health Science Center. pcDNA3.1-HBx was constructed by inserting an HBx fragment amplified from pZac2.1-HBV with HindIII/Xba I. All the plasmids used in these transfection experiments were prepared using a Large-scale Purification Kit (Beijing ComWin Biotech, China), following the manufacturer's recommended protocol. Cells were transfected with plasmid and mimics/inhibitors using lipofectamine 2000 (Thermo, Waltham, WA, USA), following the application guide of the product.

### Plasmid constructs and site-directed mutagenesis

PmirGLO vector (Promega, WI, USA) was used to construct the reporter plasmids. Genomic DNA from PLC cells was used as a template for PCR to obtain 165 bp (DNA sequence from 1–165 nt) fragment of the *AFP* 3′-UTR, which was inserted into the NheI /EcoRi sites at the downstream of firefly luciferase reporter gene to generate AFP-3′UTR. AFP-3′UTR contains putative miR-620, miR-1236 and miR-1270 binding sites.

The mutated constructs AFP-3′UTR-MU1 / AFP-3′UTR-MU2 were generated by mutating the seed match sequences (5'-GGGAAGAG-3′) / (5'-TATCTCCA-3′) of the miR-1236 / miR-620 and miR-1270 target sites in AFP-3′UTR to 5′-TTTCGTCG-3′ / 5′-GGCTCGGG-3′ using site-directed mutagenesis. The mutated construct AFP-3′UTR-MU was generated by mutating both the above seed match sequences. MiR-620, miR-1236, miR-1270 mimics and inhibitors were purchased from Suzhou Genepharma Inc. (Suzhou, China).

### Quantitative real-time PCR (qRT-PCR) and western blotting

Total RNA was isolated using the TRIzol reagent (Invitrogen) for both mRNA and miRNA analyses. Reverse transcription was routinely performed according to the manufacturer's instructions. Reverse transcription of miRNAs was carried out with commercial primers (Tiangen Biotech Co., Ltd. Beijing, China). The relative levels of AFP mRNA were examined using SYBR green qPCR (Vazyme Biotech Inc., Nanjing, China) and were normalized to levels of β-actin mRNA. For analysis of miR-620, miR-1236 and miR-1270 expression, qPCR analyses were conducted using TransStart Green qPCR SuperMix (TransGene biotech Inc., Beijing, China) and were normalized to the expression of U6. Primers used in qPCR are listed in Supplementary Table S1 (Supplementary Table S1). Relative expression was calculated using the 2^−^^ΔΔCT^ method. The specificity of the qRT-PCR primers was determined using a melting curve after amplification to show that only a single species of qRT-PCR product was amplified from the reaction. The qRT-PCR experiments were repeated at least three times. The relative concentration of *AFP* mRNA is presented as mean fold-change of samples compared with the control. Western blotting was carried out to detect AFP levels, as described previously [7]. Primary antibodies against AFP and β-actin were purchased from (Santa Cruz Biotechnology, Santa Cruz, CA, USA).

### Luciferase reporter assay

Luciferase assays were routinely performed [44]. PLC cells were transfected with all constructs and subjected to a dual luciferase reporter assay at 36 h after transfection. Firefly luciferase activity was normalized to Renilla luciferase activity. The original pmirGLO vector served as a negative control.

### Determination of viability of PLC/PRF/5 cells

The effect of icaritin on cell proliferation was assessed using a Cell Counting Kit (CCK)-8 (Dojindo Laboratories, Kumamoto, Japan). Briefly, PLC cells were adjusted to 5 × 10^4^ ml^−1^ and aliquoted into 96-well plates. The cells were treated with different concentrations of icaritin (0–40 μM) for 48 h and then with 10 μl CCK Solution Reagent for another 4 h, according to the manufacturer's protocol. The viability of cells was detected on a Universal Microplate Reader (EL X 800) at 450 nm. The cell viability after each treatment was calculated as % cell inhibition ratio= [(*A*_450_ sample-background) / (*A*_450_ control-background)] × 100%.

### 5-ethynyl-2'-deoxyuridine (EdU) proliferation assay

Proliferating PLC cells were determined using the Cell-Light™ EdU Apollo®488 *In Vitro* Imaging Kit (RiboBio Co., Ltd. Guangzhou, China), according to the manufacturer's protocol. Briefly, cells were incubated with 50 μM EdU for 2 h before fixation, permeabilization, and EdU staining. Cell nuclei were stained with 1×Hoechst 33342 for 30 min. EdU is a nucleoside analog of thymidine that is incorporated into DNA during active DNA synthesis only by proliferating cells. After incorporation, a fluorescent molecule was added that reacted specifically with EdU, making possible fluorescent visualization of proliferating cells. The fluorescent images were observed on Operetta™ (a high content imaging system) at 340 and 488 nm.

### Flow cytometry analysis for apoptosis

Flow cytometry was performed in a standard manner to determine the effect of icaritin on apoptosis. Briefly, PLC cells were treated with different concentrations of icaritin (0–40 μM) for 48 h and harvested for analysis by trypsinization, and then stained using an Annexin V/PI Apoptosis Detection Kit (Dojindo Laboratories, Kumamoto, Japan), according to manufacturer's instructions. Cell apoptosis was analyzed using a FACScalibur flow cytometer (BD Biosciences, San Jose, CA, USA). The fluorescence signals of apoptotic cells were represented by Annexin V^+^ /PI^−^ (early apoptosis) and Annexin V^+^ /PI^+^ (late apoptosis/necrosis). FITC-conjugated Annexin V can be detected by flow cytometry or by fluorescence microscopy.

### Detection of serological markers of HBV

HBV infection and the expression of HBsAg and HBeAg were determined using the KHB ELISA (enzyme linked immunosorbent assay) test kits (Shanghai Kehua Bioengineering Co. Ltd., China) according to the manufacturer's instructions. DMEM medium supernatant was collected after transfection with an HBV plasmid for 48 hours and added to the matched ELISA microplates, before adding the enzyme conjugate, washing, and detecting the chromogenic reaction. The titer of HBsAg and HBeAg were detected on a universal microplate reader (EL X 800) at 450 nm (reference wavelength 630 nm).

### Statistical analysis

Statistical analysis was carried out using SPSS version17.0, and significance was determined using a two-tailed Student's t-test. All data are represented as mean ± SD and P < 0.05 was considered statistically significant.

## SUPPLEMENTARY FIGURES AND TABLE


