# Supplementary material for: Icaritin inhibits the expression of alpha-fetoprotein in hepatitis B virus-infected hepatoma cell lines through post-transcriptional regulation
Source: Oncotarget. 2016 Nov 8;7(50):83755–66. doi: 10.18632/oncotarget.13194 (PMC5347802; doi:10.18632/oncotarget.13194)
Supplement: Supplementary file 1 [file oncotarget-07-83755-s001.pdf]

Icaritin inhibits the expression of alpha-fetoprotein in hepatitis B virus-infected hepatoma cell lines through post-transcriptional regulation

SUPPLEMENTARY FIGURES AND TABLE

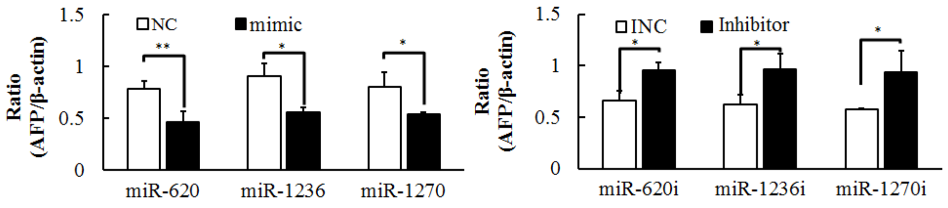

Supplementary Figure S1: Gray scale analysis of the expression of AFP in miR-620, miR-1236, miR-1270 transfected cells. These experiments were repeated at least three times. Data represents mean ± SD of three samples. *$P < 0.05$ and **$P < 0.01$ as compared with controls.

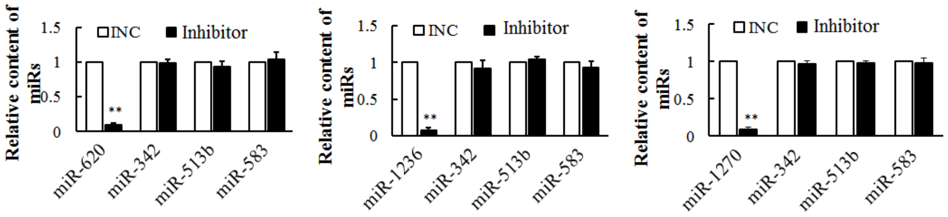

Supplementary Figure S2: Determination of the specificity of the inhibitors of miR-620, miR-1236 and miR-1270. These experiments were repeated at least three times. Data represents mean ± SD of three samples. $*P < 0.05$ and $**P < 0.01$ as compared with controls.

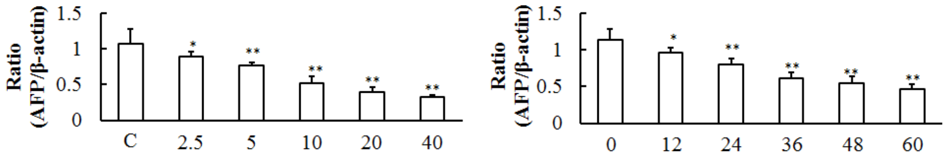

Supplementary Figure S3: Gray scale analysis of effects of icaritin on the expression of AFP. These experiments were repeated at least three times. Data represents mean ± SD of three samples. *P < 0.05 and **P < 0.01 as compared with controls.

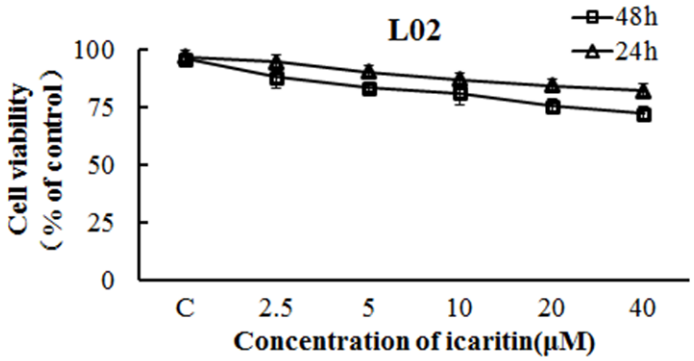

Supplementary Figure S4: Effects of icaritin on the growth of L02 normal human hepatocytes. These experiments were repeated at least three times. Data represents mean ± SD of three samples. *$P < 0.05$ and **$P < 0.01$ as compared with controls.

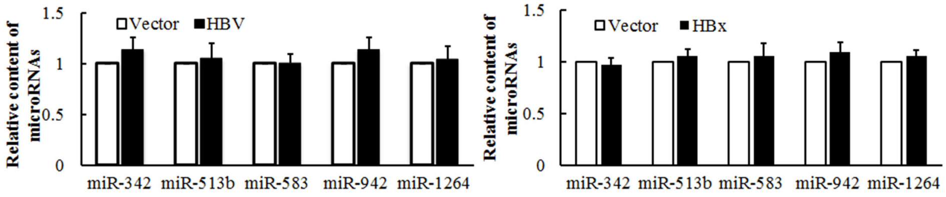

Supplementary Figure S5: Effects of HBV and HBx on the expression of miR-342, miR-513b, miR-583, miR-942 and miR-1264. These experiments were repeated at least three times. Data represents mean ± SD of three samples. $*P < 0.05$ and $**P < 0.01$ as compared with controls.

Supplementary Table S1: Sequences of oligonucleotides used for RT-qPCR and plasmid constructs assays

		Oligonucleotides sequences	Product size(bp)
**Primers for RT-qPCR**			
AFP	Sense	5'- CCAACAGGAGGCCATGCTT -3'	61
	Antisense	5'- GAATGCAGGAGGGACATATGTTT -3'	
β-actin	Sense	5'- CCAACCGCGAGAAGATGA -3'	97
	Antisense	5'- CCAGAGGCGTACAGGGATAG -3'	
U6	Sense	5'- GCTTCGGCAGCACATATACTAAAAT -3'	
**Primers for plasmid constructs**			
HBx	Sense	5'- CCCAAGCTTATGGCTGCTCGGGTGTGCT -3'	
	Antisense	5'- GCTCTAGAGCCAGCTTGGAGGCTTGAACAG -3'	
AFP-3'UTR	Sense	5'- CGGCTAGCATTACTTCAGGGGAAG -3'	
	Antisense	5'- CGGAATTCGAAAAGGAAACATTTGG -3'	
AFP-3'UTR-MU	Sense	5'- GGCTAGCATTACTTCAGTTTCGTCGAAGAC -3'	
	Antisense	5'- GGAATTCGGAAACATTCCCGAGCGTTTTAT -3'	
AFP-3'UTR-MU1	Sense	5'- GGCTAGCATTACTTCAGTTTCGTCGAAGAC -3'	
	Antisense	5'- CGGAATTCGAAAAGGAAACATTTGG -3'	
AFP-3'UTR-MU2	Sense	5'- CGGAATTCGAAAAGGAAACATTTGG -3'	
	Antisense	5'- GGAATTCGGAAACATTCCCGAGCGTTTTAT -3'	
